# PERK/eIF2α pathway affected the thyroid hormone synthetic in hypertensive disorders of pregnancy rats

**DOI:** 10.3389/fendo.2025.1552065

**Published:** 2025-08-13

**Authors:** Congrong Wu, Maomao Sun, Yue He, Jie Jiang, Luran Wang, Yanni Wang, Yonghui Yu

**Affiliations:** ^1^ Department of Pediatrics, Shandong Provincial Hospital affiliated to Shandong First Medical University, Jinan, China; ^2^ Weifang (W. F.) Maternal and Health Hospital, Weifang, China; ^3^ Department of Public Health and Health Management, Shandong First Medical University, Jinan, China

**Keywords:** hypertensive disorders of pregnancy, subclinical hypothyroidism during pregnancy, thyroid hormone synthesis, endoplasmic reticulum stress, PERK/eIF2α pathway

## Abstract

**Background:**

Clinical research has identified a correlation between hypertensive disorders of pregnancy (HDP) and subclinical hypothyroidism during gestation. But the potential influence of HDP on thyroid hormone synthesis remains undetermined.

**Aims:**

This study aims to elucidate the impact of HDP on thyroid hormone synthesis and to delineate the underlying mechanisms.

**Methods:**

20 pregnancy SD rats were stratified at random into the HDP group and the Control group. The HDP group was subjected to NG-Nitro-L-arginine-methylester administration from gestational days 13 to 20, while the Control group received saline. Subsequent assessments encompassed serum FT4, FT3, and TSH concentrations, morphological examination of the thyroid, as well as quantification of essential proteins pivotal to thyroid hormone synthesis and markers indicative of endoplasmic reticulum stress.

**Results:**

The HDP group exhibited a statistically significant augmentation in serum TSH concentrations (*p*<0.05), while FT3 and FT4 levels manifested no discernible statistical variations. H&E staining highlighted a pronounced hyperplasia of the follicular epithelial cells and a diminution in the follicle lumen area. Electron microscopy unveiled pronounced endoplasmic reticulum markedly swelling and expansion within the HDP group. Molecular evaluations revealed a decrement in Tg expression within thyroid tissue, concomitant with an upregulated expression of p-PERK, P-eIF2α, and ATF4.

**Conclusion:**

This investigation suggests that HDP might modulate Tg expression within thyroid tissue, possibly mediated through the PERK/eIF2α signaling cascade. This perturbation may compromise thyroid hormone synthesis, thereby predisposing pregnant rats to subclinical hypothyroidism.

## 1 Introduction

Hypertensive disorders of pregnancy (HDP) are one of the most prevalent complications of pregnancy, with an alarming global prevalence of 116 per 100,000 women within the reproductive age range (1). HDP not only escalates the likelihood of maternal mortality and morbidity but also predisposes the risk of postpartum disease including hypertension, cardiovascular disorders, and chronic kidney disease (2). Furthermore, fetuses subjected to HDP are at an augmented risk for intrauterine growth restriction, premature birth, and reduced birth weight (3). Notably, human studies demonstrate similar HDP-associated thyroid dysfunction (4, 5), underscoring the translational relevance of our mechanistic findings. Importantly, these complications result in a large disease burden for pregnant individuals and their offspring.

Thyroid hormones play an indispensable role in ensuring optimal fetal growth and development in gestation. Subclinical hypothyroidism in pregnancy has been linked to maternal and fetal outcomes encompassing spontaneous abortion, gestational diabetes mellitus, preterm delivery, and attenuated Intelligence Quotient in the offspring (6, 7). It is noteworthy that growing a body of studies have reported a potential association between HDP and an elevated propensity for subclinical hypothyroidism during pregnancy (4, 5, 8). Further, the role of endoplasmic reticulum stress (ER stress) in the pathogenesis of hypothyroidism has been substantiated in previous research endeavors (8–10). While prior studies have established clinical correlations between HDP and thyroid dysfunction, the mechanistic role of ER stress, particularly the PERK/eIF2α pathway, in disrupting thyroid hormone synthesis remains unexplored. Our study addresses this gap by demonstrating how HDP-induced ER stress impairs thyroglobulin (Tg) processing, providing a molecular explanation for subclinical hypothyroidism in HDP.

The current investigation emulated the HDP model in SD rats through subcutaneous injection of NG-Nitro-L-arginine-methylester (L-NAME) (11, 12). By assessing thyroid function, scrutinizing thyroid morphology, and quantifying the expression profiles of pivotal proteins for thyroid hormone synthesis and the sensitive indicators of ER stress to verify if HDP exerts its influence on thyroid hormone synthesis via ER stress while exploring the potential underlying mechanisms.

## 2 Materials and methods

### 2.1 Animals

Twenty adult female Sprague-Dawley rats, 10–12 weeks old, weighing between 280–300 g, were procured from Beijing Weitong Lihua Laboratory Animal Technology Co., Ltd, Beijing, China. The rats were acclimatized for one week under controlled environmental conditions (21 ± 2 °C temperature; 38% relative humidity; and a 12-hour light/12-hour dark cycle), adaptive feeding for one week. They were provided with standard laboratory chow and had unrestricted access to water. Sample sizes for Western blot (n=3–4) were based on effect sizes observed in prior studies of ER stress in thyroid models (8, 13). While these group sizes detected significant differences, future studies will include power calculations to further validate robustness. All experimental procedures complied with international standards for laboratory animal care and were endorsed by the Animal Ethics Committee of the Shandong Provincial Hospital (Approval No. 2022 - 067).

### 2.2 Experimental protocol

Following successful mating, the presence of spermatozoa in the vaginal smear was considered on day one of gestation (GD1). Then, the pregnant rats were randomized into two experimental cohorts: the Control group (n =10) and the HDP group (n =10). Rats in the HDP group were administered daily subcutaneous injections of L-NAME (N109211, Aladdin) from GD13 to GD20 at a concentration of 250 mg/kg/d. In parallel, the Control group was given equivalent volumes of saline subcutaneously. On GD21, all rats were euthanized to procure thyroid tissue samples and blood. The establishment of the HDP model followed previous work (13). In this study, a completely blinded procedure was implemented for all outcome evaluations (including histological, ELISA and Western blot analyses). The detailed schedule of the experiment is shown in **Supplementary Figure 1**.

### 2.3 Evaluation of blood pressure and urinary protein concentration

Symptoms indicative of HDP were assessed on GD 10, GD 14, and GD 20. The tail-cuff noninvasive method was employed to measure systolic blood pressure (SBP), mean arterial blood pressure (MBP), and diastolic blood pressure (DBP). Concurrently, urinary protein concentrations on GD10 and GD20 were quantified utilizing a BCA assay kit (BL521A, Beyotime).

### 2.4 Thyroid hormone analysis

The concentration of TSH was determined through an ELISA assay (CEA463Ra, Cloudcloning), while FT4 and FT3 levels were utilized chemiluminescence assays at the Department of Clinical Laboratory, Shandong Provincial Hospital Affiliated to Shandong First Medical University. All assays were conducted in strict accordance with the manufacturer’s guidelines.

### 2.5 Histological and electron microscopy analyses

The thyroid lobes were harvested and fixed with 4% paraformaldehyde for 24 hours, the samples underwent gradient dehydration, were embedded in paraffin, sectioned (4 μm thickness), and subsequently stained with hematoxylin and eosin (H&E) for light microscopic examination. The other thyroid lobes were preserved in 3% glutaraldehyde for electron microscopic evaluation. For each animal, three non-consecutive thyroid sections (4 μm) were analyzed at 20× magnification. Follicular area and number were quantified using ImageJ (v1.53) with uniform thresholding (Huang algorithm) across all images. Data represent averages from 5 fields per section.

### 2.6 RT-qPCR analysis

Total RNA was isolated using Trizol reagent (AG21102, AG) and reverse transcription was performed with the Evo M-MLV RT Mix Kit (AG11728, AG). The resulting cDNA samples were subject to qPCR employing the SYBR Green PCR Master Mix (AG11701, AG) on a Light Cycler 480 instrument (Roche, Germany). Gene expression levels were normalized against β-actin. Primer sequences are shown in **Supplementary Table 1**.

### 2.7 Western blotting analysis

Thyroid tissues were lysed using RIPA buffer supplemented with protease and phosphatase inhibitors (Bimake, China). Post-denaturation, proteins underwent SDS-PAGE and were subsequently electrotransferred to a polyvinylidene fluoride (PVDF) membrane, after being blocked in 5% skim milk in TBS-T, and incubated overnight at 4°C with specific primary antibodies. This was followed by incubation with horseradish peroxidase-conjugated secondary antibodies. Detection of protein bands was achieved through electrochemiluminescence (36208ES60, Yeason Biotech), and visualized using a ChemiDoc system (Bio-Rad, v3.1). Densitometric analysis of protein bands was performed using ImageJ software (NIH, v1.53).

### 2.8 Statistical analysis

A *post-hoc* power analysis (G*Power 3.1) confirmed adequate sample size (n=10 per group) to detect effect sizes ≥1.5 (α=0.05, power=0.8) based on preliminary TSH and Tg data. All data were analyzed utilizing SPSS version 26.0 and are presented as mean ± SEM. The Shapiro-Wilk procedure was used for the comprehensive normality test, which confirmed that all datasets conformed to the assumption of normal distribution (p > 0.05). The F-test confirmed the homogeneity of variance among each group. Comparisons between groups were made using a Student’s T-test. Statistical significance was set at a p-value of < 0.05.

## 3 Results

### 3.1 Thyroid dysfunction in HDP rats

**Figure 1:**
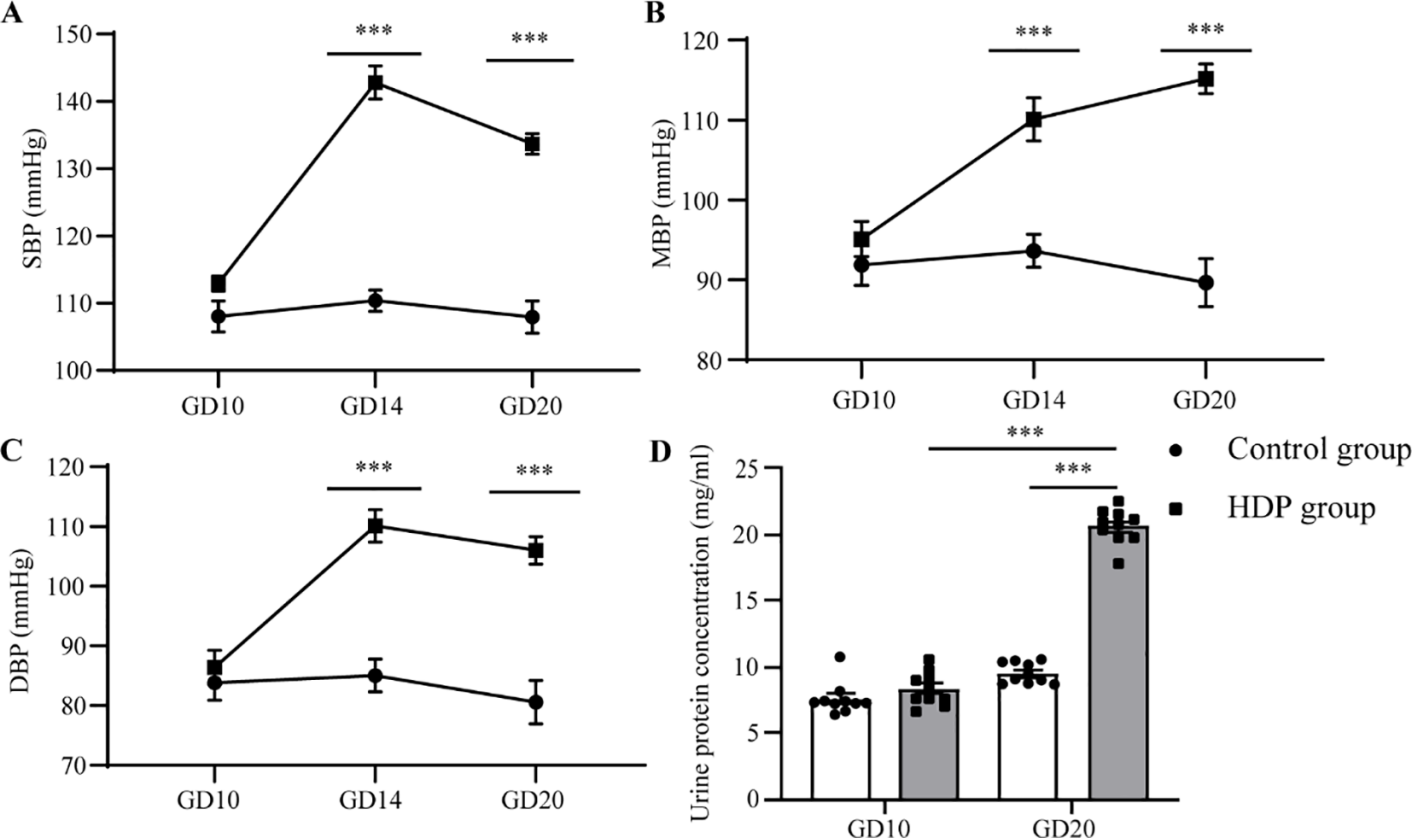
Changes in blood pressure and urinary protein in HDP rats. Rats in the control group and the HDP group were subcutaneously injected with normal saline and L-NAME respectively at a dose of 250 mg/kg/d, from GD13 to GD20. **(A)** SBP of GD10, GD14, and GD20 (n=10); **(B)** MBP for GD10, GD14, and GD20 (n=10); **(C)** DBP on GD10, GD14, and GD20 (n=10); **(D)** The influence of urinary protein on GD10 and GD20 (n=10). The error bar represents the average value ± SEM. ***p<0.001.

**Figure 2:**
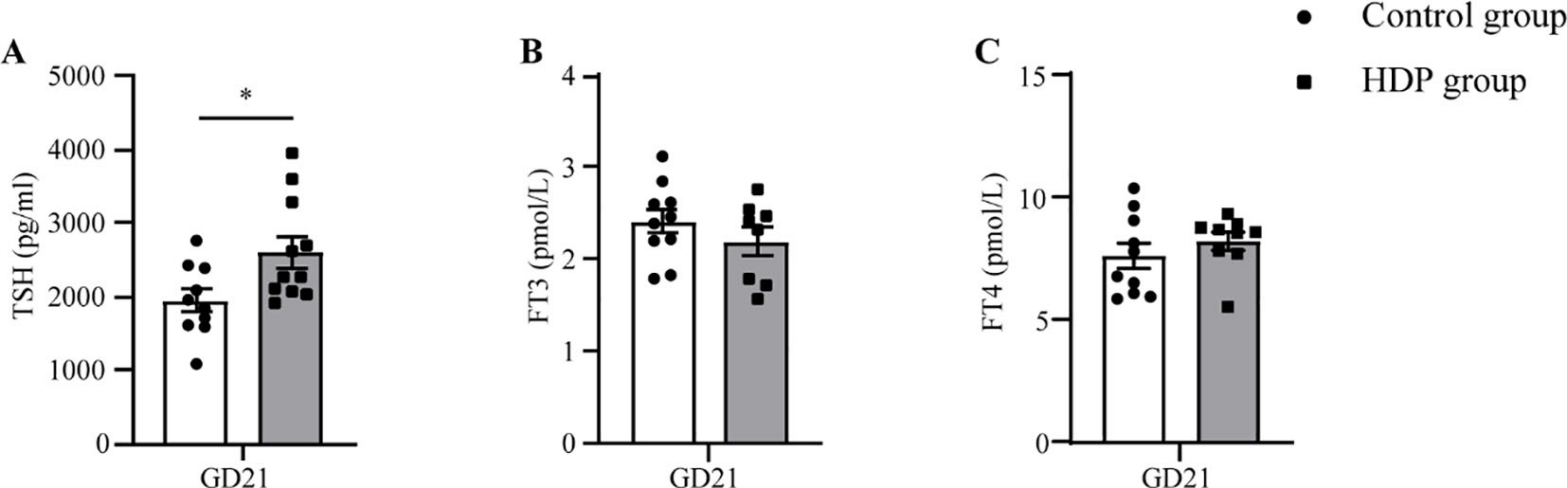
Serum hormone concentrations in HDP rats. **(A)** Serum TSH on GD21 (n=10); **(B)** Serum FT3 on GD21 (n=7-10); **(C)** Serum FT4 on GD21 (n=7-10). Error bars represent the mean ± SEM. *p<0.05.

At GD10, no discernible differences in SBP, MBP, and DBP were identified between the two cohorts. Following the administration of L-NAME via subcutaneous injection, a significant augmentation in SBP, MBP, and DBP was evident in the HDP group on GD14 and GD20 (p < 0.001) (**Figures 1A–C**). The urinary protein concentration in the HDP group surpassed that of the Control group on GD20 (p < 0.001) (**Figure 1D**). Evaluation of thyroid function elucidated that HDP rats manifested elevated TSH concentrations (p < 0.05) (**Figure 2A**), while FT3 and FT4 levels exhibited no different between the groups (**Figures 2B, C**). The early-born offspring of HDP rats have intrauterine growth restriction, as shown in **Supplementary Table 2**.

### 3.2 HDP induced the decrease of Tg

**Figure 3:**
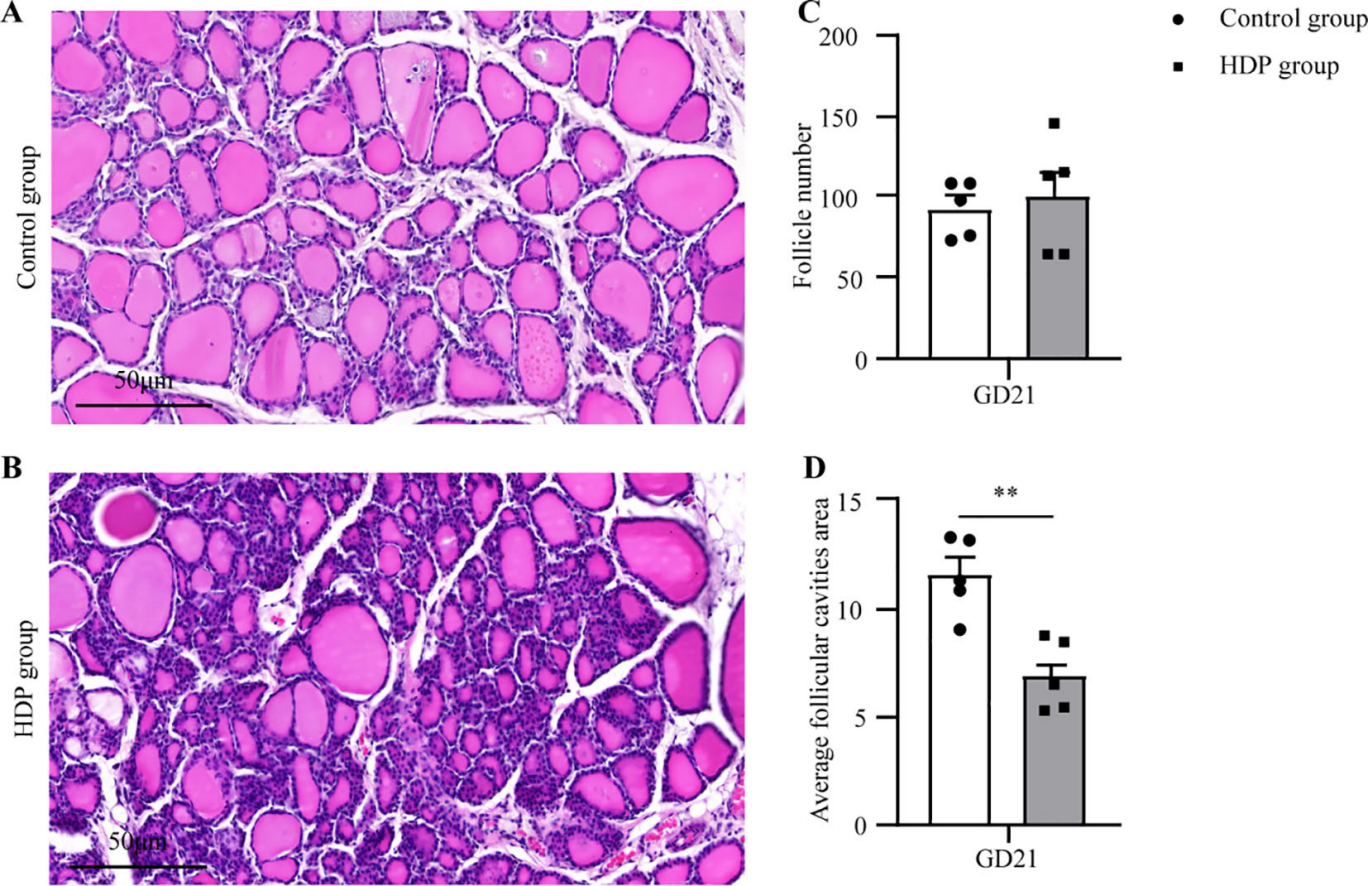
Thyroid histopathology. **(A, B)** Representative microphotographs of thyroid glands from a Control group and HDP group rat (n=5, 20X objective, Bar = 50μm); **(C)** The follicle number from Control group and HDP group rats (n=5); **(D)** The Average follicular cavities area from Control group and HDP group rats (n=5). Error bars represent the mean ± SEM. **p<0.01.

**Figure 4:**
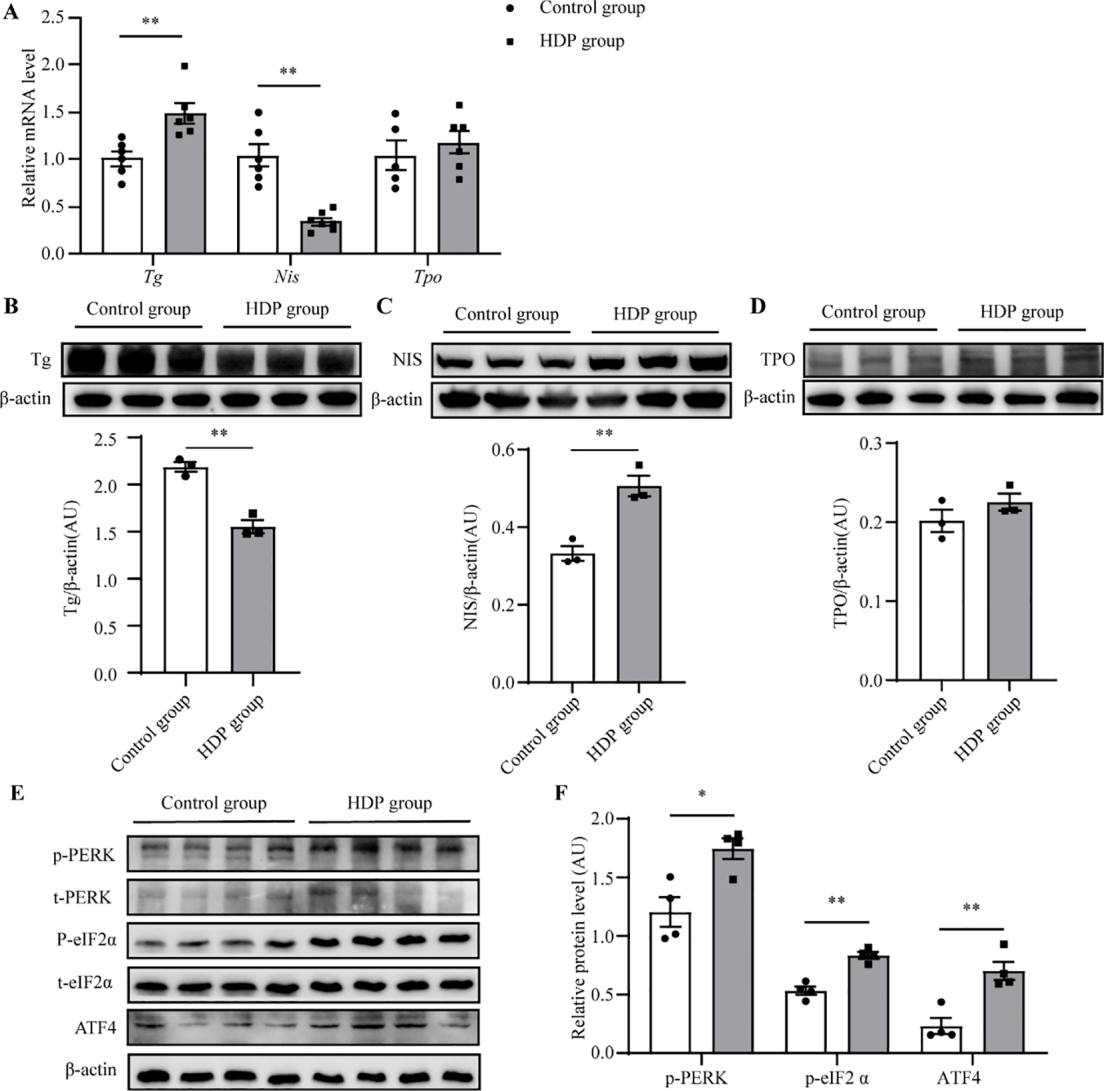
HDP induces a decrease in Tg protein. **(A)** Expression levels of Tg, Tpo and Nis mRNA in the thyroid glands of rats in the control group and the HDP group (n=6); **(B)** Western blot analysis of Tg protein level in Control group and HDP group rats (n=3); **(C)** Western blot analysis of NIS protein level in Control group and HDP group rats (n=3); **(D)** Western blot analysis of TPO protein level in Control group and HDP group rats (n=3); **(E, F)** Western blot analysis of p-PERK, p-eIF2α, and ATF4 activation level in Control and HDP group (n=4). Values were quantified by densitometry, p-PERK and p-eIF2a were normalized with their respective total protein, while Tg, NIS, TPO and ATF4 were normalized with β-actin. Error bars represent the mean ± SEM. *p<0.05 **p<0.01.

Histopathological assessment under light microscopy unveiled distinct thyroid morphological alterations (**Figures 3A, B**). The Control group displayed uniformly distributed follicles of medium dimension, with larger follicles localized peripherally, and epithelial cells flat or short cubic. The HDP group epithelial cell morphology and follicular counts mirrored that of the Control group (**Figure 3C**). Remarkably, the HDP group exhibited follicular epithelial proliferation alongside reduced follicular luminal area (*p* < 0.01) (**Figure 3D**). Subsequent analyses of pivotal proteins central to thyroid hormone biosynthesis at transcriptional revealed an upregulation of *thyroglobulin (Tg)* in the HDP group, a contrasting downregulation of *sodium/iodide symporter (Nis)* (*p* < 0.05), and a analogously *thyroid peroxidase (Tpo)* expression across groups (**Figure 4A**). Western blot assessments elucidated a reduction in Tg (*p* < 0.001) (**Figure 4B**), and an enhancement of NIS in the HDP group (*p* < 0.01) (**Figure 4C**), with TPO expression remaining unchanged (**Figure 4D**).

### 3.3 ER stress in the thyroid of HDP rats

**Figure 5:**
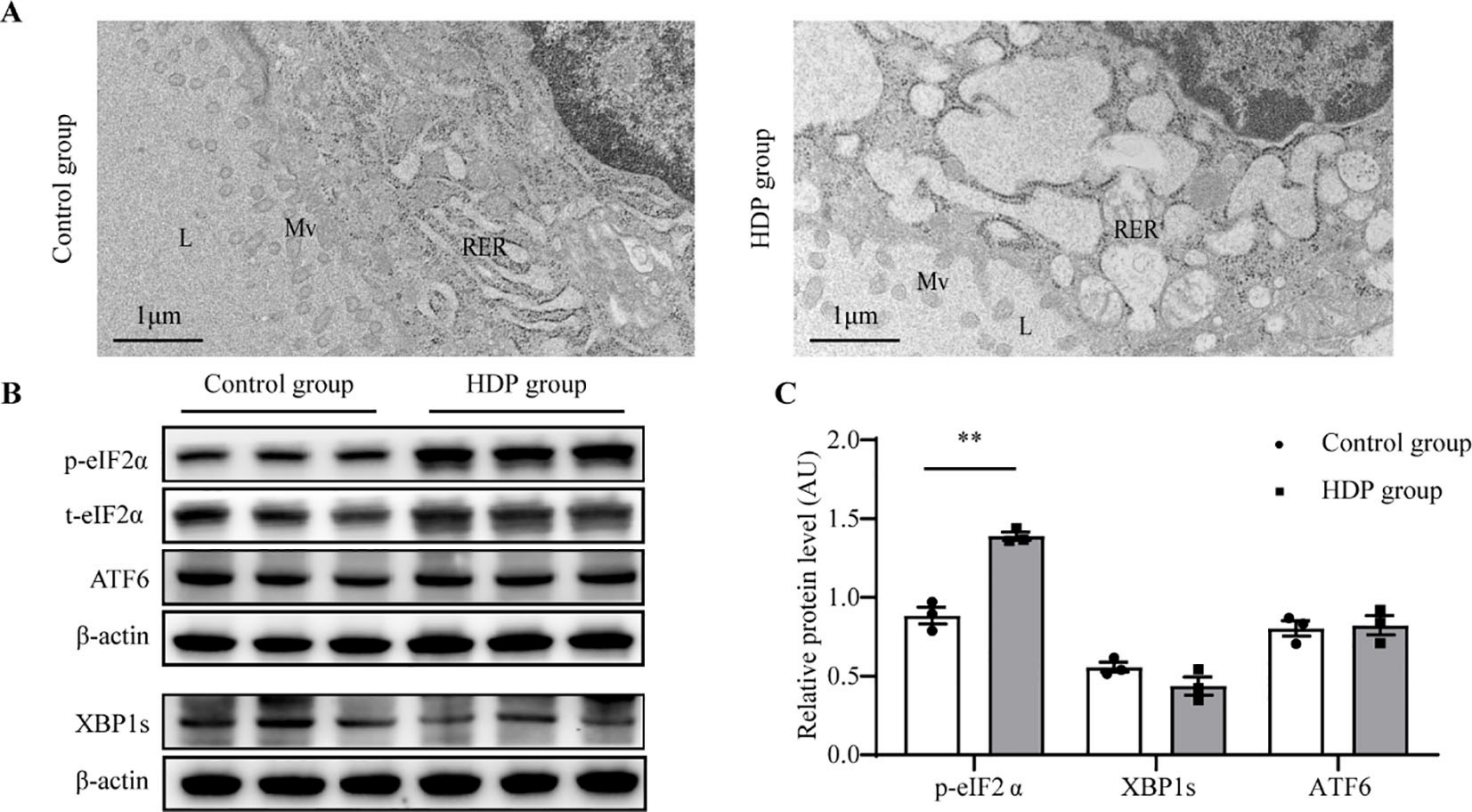
ER stress in the thyroid of HDP rats. **(A)** Representative electron micrographs of thyroid glands from a Control group and HDP group rat. L, lumen of the follicles; Mv, microvilli; RER, rough endoplasmic reticulum; N, nucleus; **(B, C)** Western blot analysis of p-eIF2α, XBP1s and ATF6 activation level in Control group and HDP group (n=3). Values were quantified by densitometry, p- eIF2α were normalized with its total protein, while XBP1s and ATF6 were normalized with β-actin; Error bars represent the mean ± SEM.** p<0.01.

Tg requires complex modifications to function, which relies on endoplasmic reticulum (ER) homeostasis. Firstly, the ultrastructure of follicular epithelial cells showed significant expansion and swelling of the ER in the HDP group, indicating the pathological changes in ER function (**Figure 5A**). Then, we asked whether HDP induced the ER stress by detecting the three unfolded protein response (UPR) signaling pathways which have been characterized in metazoans: PRKR-like ER kinase (PERK)–eukaryotic translation initiation factor 2α (eIF2α), inositol requiring protein 1α (IRE1α)–X-box-binding protein 1 (XBP1), and activating transcription factor (ATF)6α. Phosphorylated eIF2α (p-eIF2α), transcriptionally active XBP1 (XBP1s) and ATF6α are the sensitive indicator of activation of the PERK, IRE1 and ATF6α respectively (14–16). The HDP group exhibited elevated p-eIF2α levels (*p* < 0.01), while XBP1s and ATF6 expression remained invariant (**Figures 5B, C**). This indicated activation of the PERK/eIF2α UPR arm, with IRE1 and ATF6 branches remaining dormant. Subsequent validation of the PERK/eIF2α pathway demonstrated upregulated expression of p-PERK, p-eIF2α and ATF4 in the HDP group (**Figures 4E, F**). These intimated that HDP-mediated diminution of Tg protein is orchestrated through the PERK/eIF2α cascade.

## 4 Discussion

This study demonstrates that HDP induce subclinical hypothyroidism through ER stress-mediated impairment of Tg processing in a well-characterized L-NAME rat model. Our findings reveal that HDP rats developed the characteristic triad of hypertension, proteinuria and elevated TSH with normal FT3/FT4 levels, faithfully recapitulating the clinical presentation of subclinical hypothyroidism observed in human pregnancies complicated by hypertensive disorders. The diagnostic criteria employed align with established rodent models of thyroid dysfunction while acknowledging the inherent physiological differences between species that necessitate caution in direct clinical extrapolation (14). Histopathological examination uncovered significant structural remodeling of thyroid tissue, featuring reduced follicular luminal area and pronounced epithelial hyperplasia - morphological changes consistent with compensatory adaptation to impaired hormone synthesis.

Most remarkably, we observed a striking discordance between elevated Tg mRNA expression and significantly reduced Tg protein levels, suggesting post-transcriptional regulation through ER stress pathways. This interpretation is supported by ultrastructural evidence of ER dilatation and molecular markers of unfolded protein response (UPR) activation, particularly the PERK/eIF2α/ATF4 axis which showed 2–3 fold upregulation in HDP rats compared to controls (15–17). While our data primarily implicate the PERK/eIF2α pathway, minor non-significant trends in IRE1/XBP1 and ATF6 markers may warrant further investigation in larger cohorts. The selective activation of this UPR branch, with unchanged IRE1/XBP1 and ATF6 pathways, likely reflects the unique proteostatic demands of thyroid follicular cells that must process massive quantities of Tg - a complex 660kDa glycoprotein requiring extensive post-translational modification (18–21). Our observations parallel recent findings in metabolic disorder models, where chronic ER stress similarly induced PERK-mediated Tg suppression, suggesting this may represent a conserved pathway of thyroid dysfunction across diverse pathological stimuli (8, 13, 15, 16). The inverse relationship between Tg downregulation and sodium/iodide symporter (NIS) upregulation points to an elegant compensatory mechanism maintaining iodide uptake capacity despite impaired Tg utilization, as recently characterized in other systems (22–25). While these findings provide important mechanistic insights, several limitations must be acknowledged.

While GD21 captures peak HDP effects, this single time-point precludes analysis of dynamic changes in thyroid function across gestation. Future studies should incorporate earlier time points (e.g., GD14, GD18) to delineate the progression of ER stress and thyroid dysfunction (26). Sample size constraints, while adequate for detecting our primary endpoints, may limit power for secondary analyses. Most importantly, species differences in placental architecture and thyroid regulation necessitate validation in human-derived models. We are currently addressing these limitations through expanded longitudinal studies incorporating serial thyroid assessments and parallel experiments in human thyroid organoids. The PERK/eIF2α pathway could serve as a biomarker for early detection of thyroid dysfunction in HDP patients, enabling timely intervention. Therapeutic targeting of ER stress with agents like tauroursodeoxycholic acid (TUDCA) may mitigate thyroid impairment, offering a novel strategy for high-risk pregnancies. These findings underscore the need for routine thyroid monitoring in HDP to prevent adverse maternal and fetal outcomes. The conserved nature of ER stress responses across tissues and species lends credence to the broader relevance of these findings beyond pregnancy-related hypertension. While our data implicate PERK/eIF2α-mediated Tg suppression, direct evidence of Tg degradation (e.g., pulse-chase assays) or ubiquitination under ER stress would strengthen mechanistic claims. Such experiments represent a critical next step to fully elucidate Tg handling in HDP (14, 20). In addition, although our main focus was on the pathophysiology of the maternal thyroid, we enhanced the validity of the pregnancy model by incorporating neonatal growth parameters (birth length and weight) of preterm infants. A detailed assessment of thyroid function in the offspring has been reported in our previous publication (27).

In conclusion, our research indicates that HDP weakens the expression of Tg in thyroid tissue by regulating the PERK/eIF2α pathway, ultimately leading to impaired thyroid hormone synthesis and the occurrence of subclinical hypothyroidism, providing a framework for the development of biomarkers and targeted therapeutic intervention.

## Data Availability

The original contributions presented in the study are included in the article/**Supplementary Material**, further inquiries can be directed to the corresponding author/s.
