# Correction: PERK/eIF2a pathway affected the thyroid hormone synthetic in hypertensive disorders of pregnancy rats

**DOI:** 10.3389/fendo.2025.1688844

**Published:** 2025-09-04

**Authors:** Congrong Wu, Maomao Sun, Yue He, Jie Jiang, Luran Wang, Yanni Wang, Yonghui Yu

**Affiliations:** ^1^ Department of Pediatrics, Shandong Provincial Hospital affiliated to Shandong First Medical University, Jinan, China; ^2^ Weifang (W. F.) Maternal and Health Hospital, Weifang, China; ^3^ Department of Public Health and Health Management, Shandong First Medical University, Jinan, China

**Keywords:** hypertensive disorders of pregnancy, subclinical hypothyroidism during pregnancy, thyroid hormone synthesis, endoplasmic reticulum stress, PERK/eIF2α pathway

Affiliations “Department of Pediatrics, Shandong Provincial Hospital affiliated to Shandong First Medical University, Jinan, China and Department of Public Health and Health Management, Shandong First Medical University, Jinan, China” were erroneously given as “Department of Neonatology, Shandong Provincial Hospital Affiliated to Shandong First Medical University, Jinan, China and Departments of Neonatology, Shandong Provincial Hospital, Shandong University, Jinan, China”.

The original version of this article has been updated.

