# Supplementary figures and images for: PERK/eIF2α pathway affected the thyroid hormone synthetic in hypertensive disorders of pregnancy rats
Source: Front Endocrinol (Lausanne). 2025 Aug 13;16:1552065. doi: 10.3389/fendo.2025.1552065 (PMC12380578; doi:10.3389/fendo.2025.1552065)

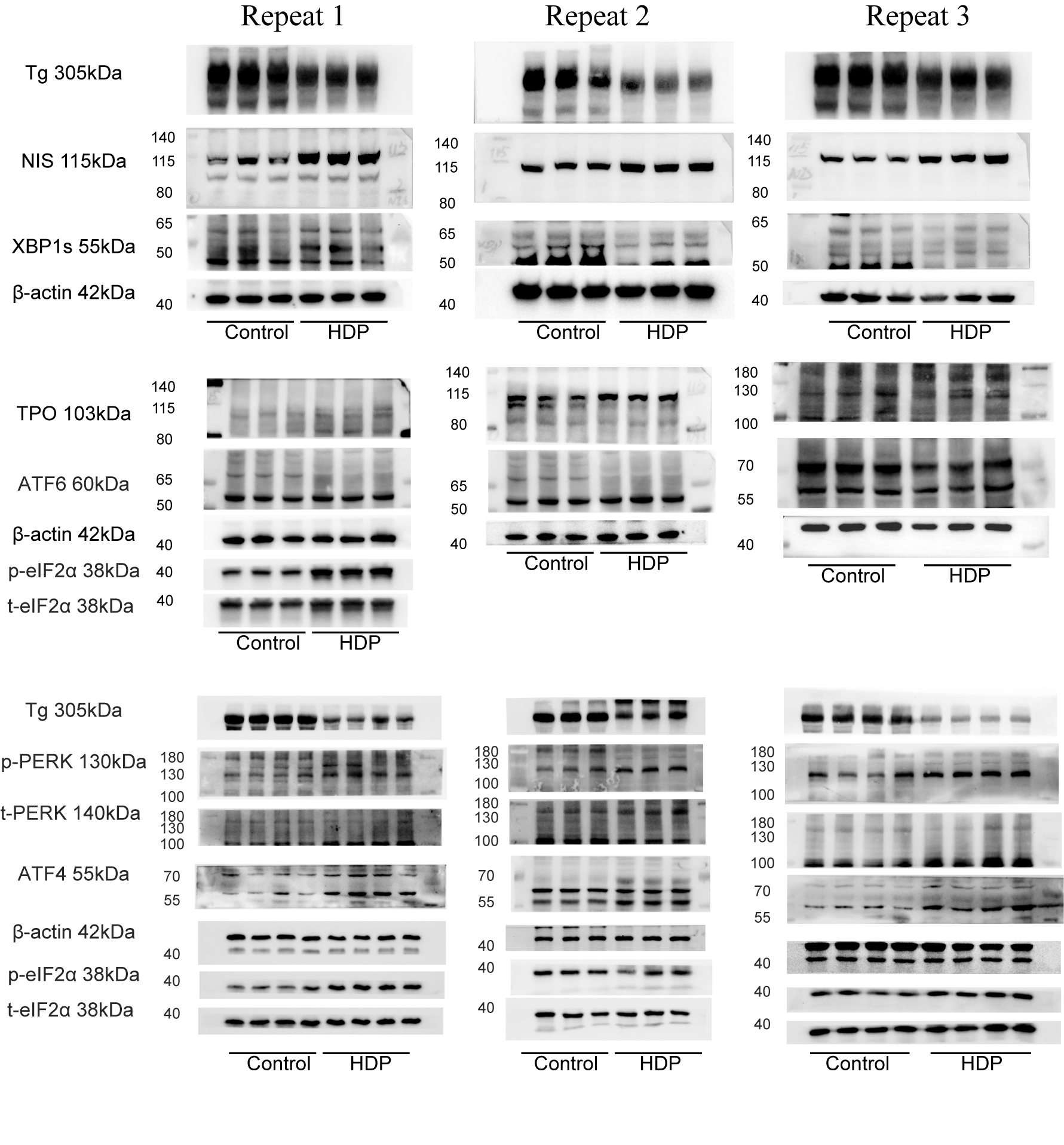

Supplement: Supplementary file 1 [file Image1.tif]

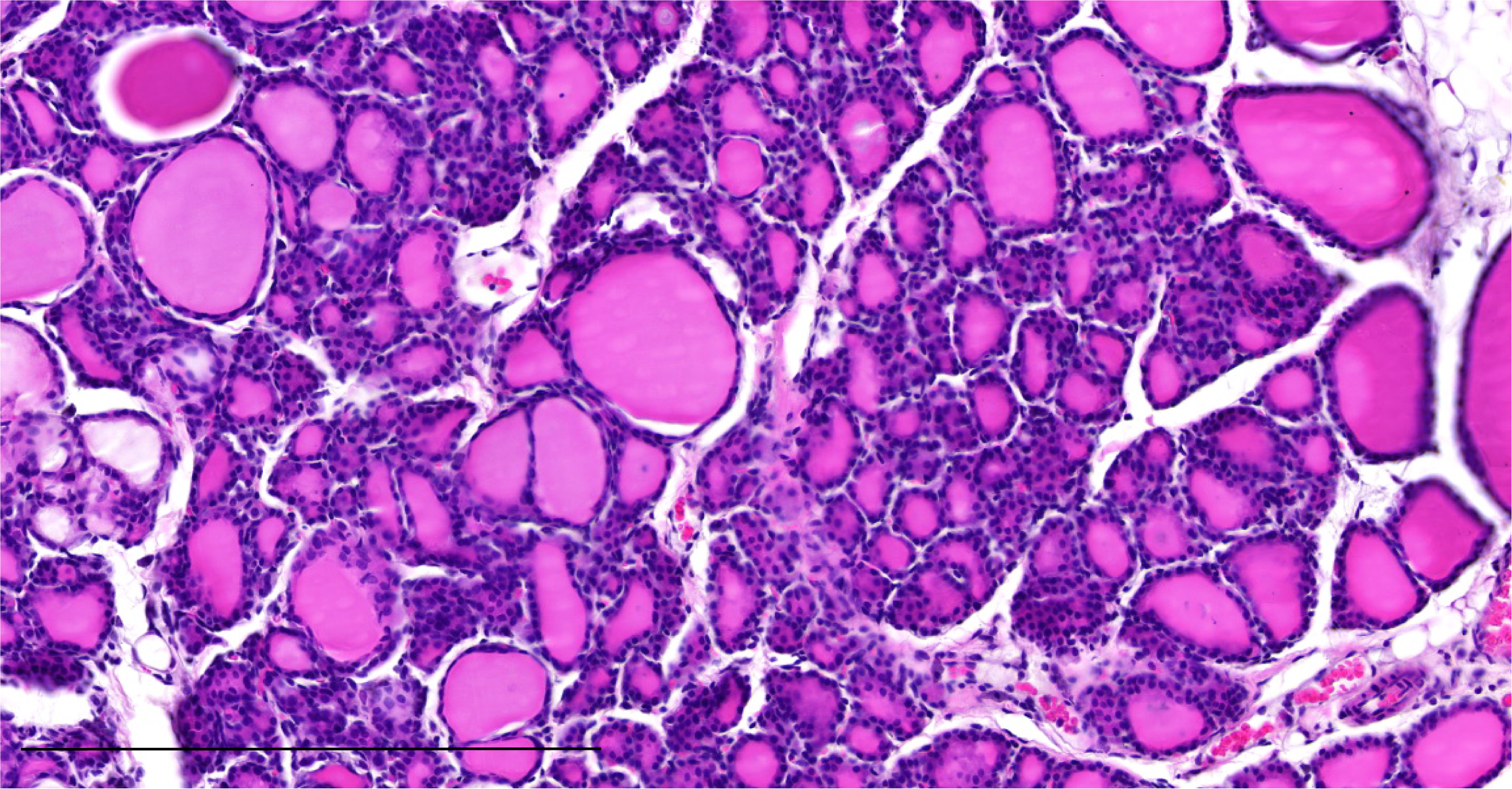

Supplement: Supplementary file 2 [file Image2.tif]

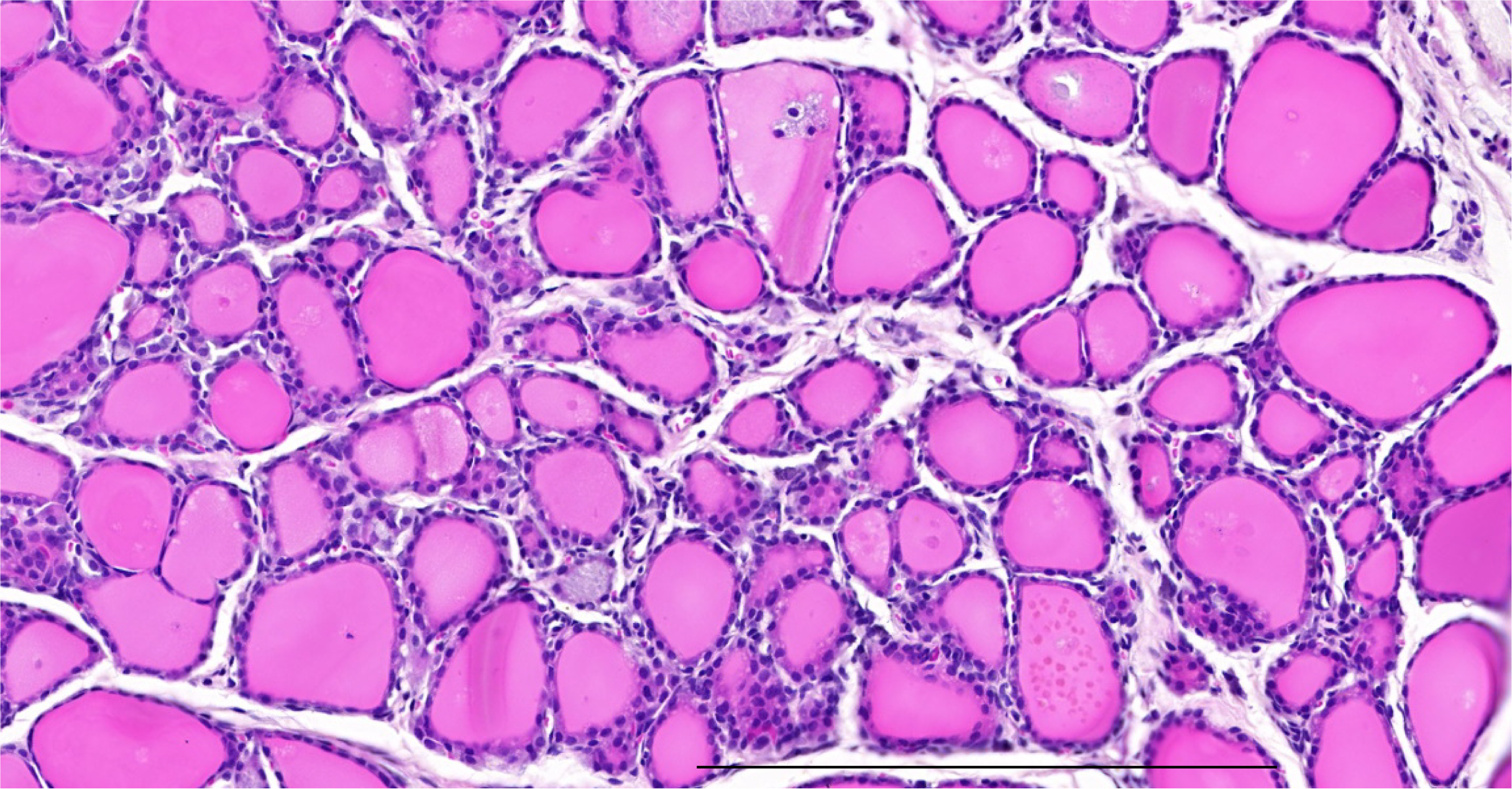

Supplement: Supplementary file 3 [file Image3.tif]

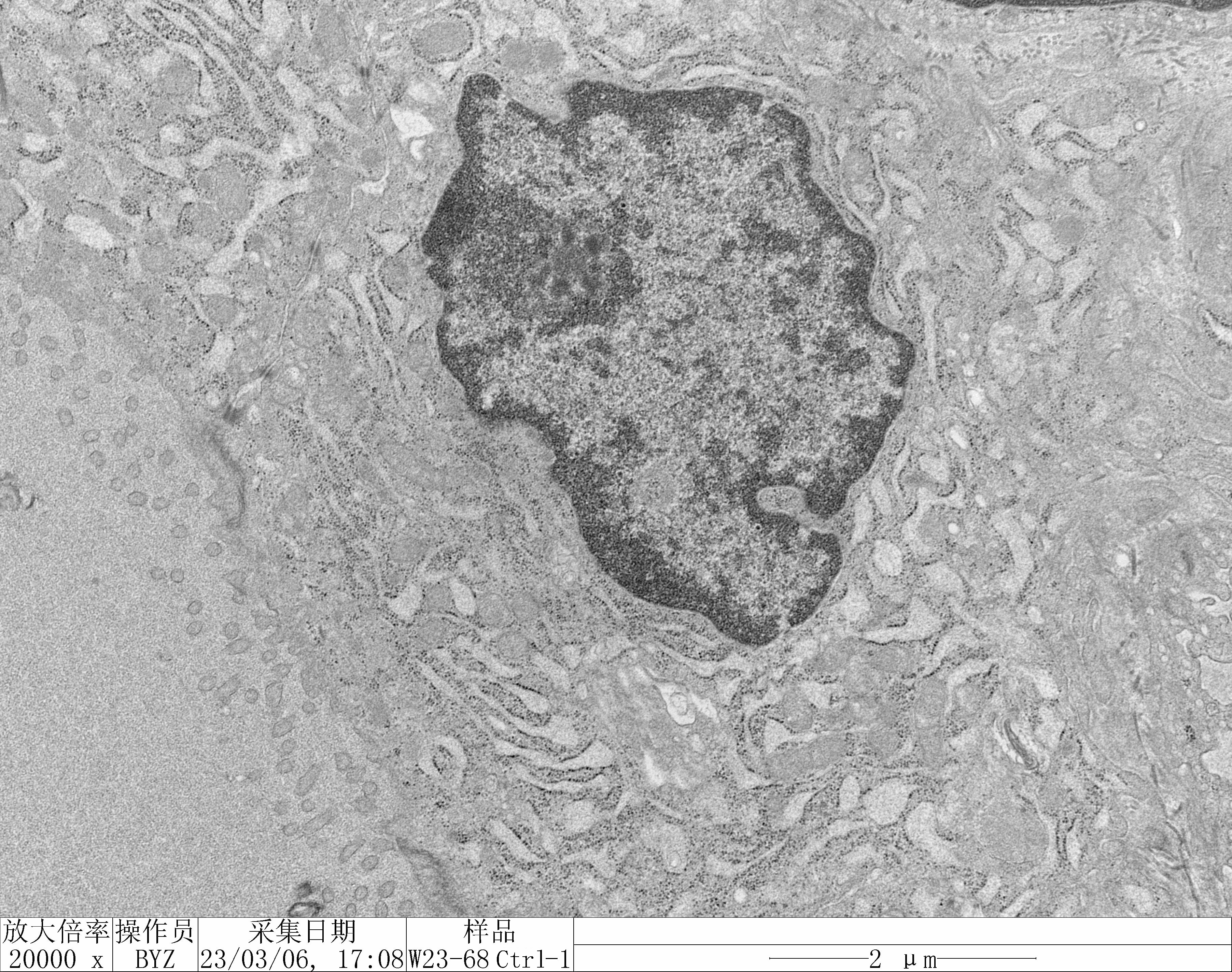

Supplement: Supplementary file 4 [file Image4.jpeg]

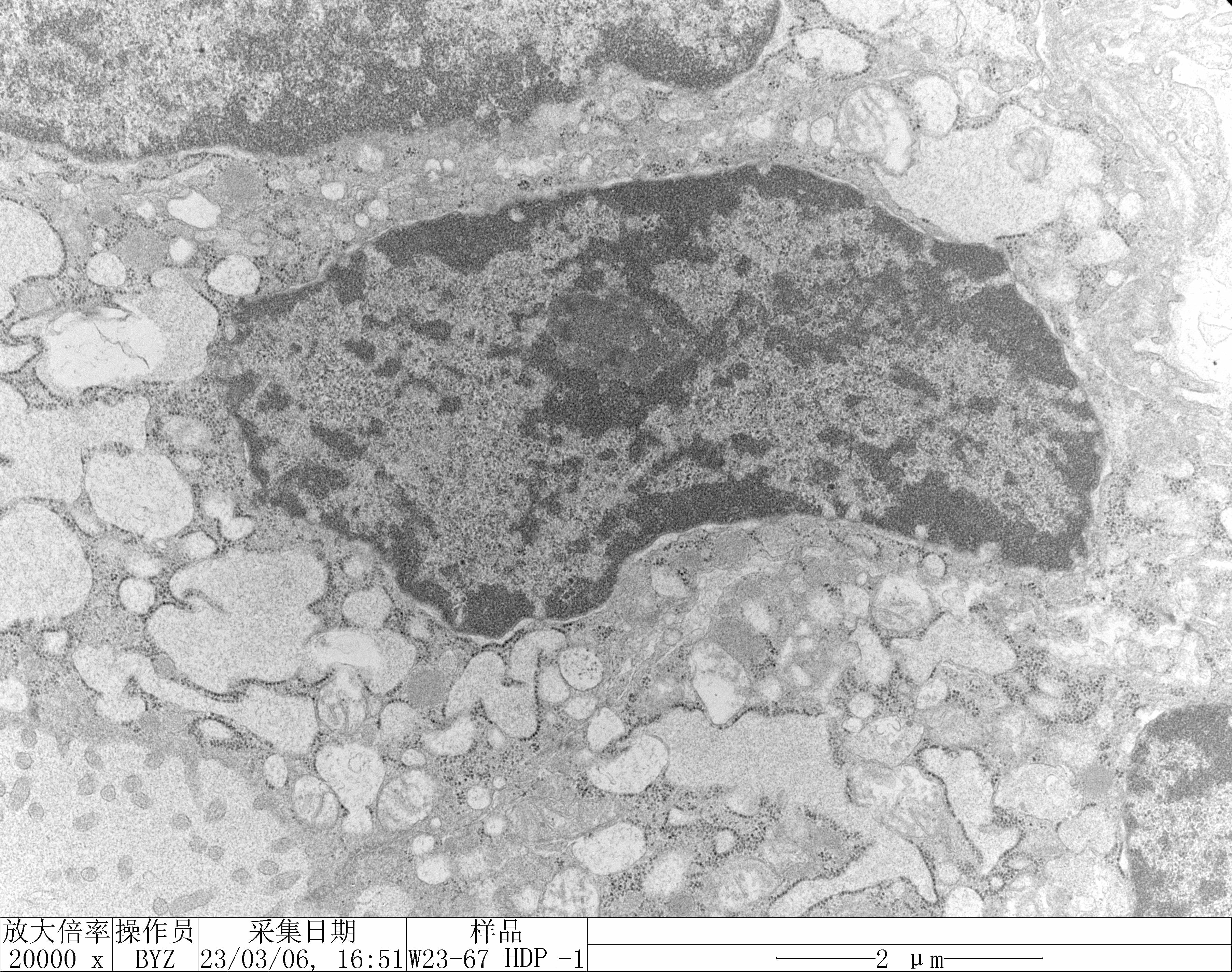

Supplement: Supplementary file 5 [file Image5.jpeg]
