# Supplementary material for: PERK/eIF2α pathway affected the thyroid hormone synthetic in hypertensive disorders of pregnancy rats
Source: Front Endocrinol (Lausanne). 2025 Aug 13;16:1552065. doi: 10.3389/fendo.2025.1552065 (PMC12380578; doi:10.3389/fendo.2025.1552065)
Supplement: Supplementary file 6 [file Table1.docx]

**Supplementary**

Table 1. Primer sequences.

| Gene | Primer sequence (5' - 3') | | Product bp |
| --- | --- | --- | --- |
| Rat |  |  |  |
| *Tg* | Forward | ACCCCAACACCTCCTCAAAT | 129 |
|  | Reverse | ACCTCCACTGCCTTCCATCT |  |
| *Tpo* | Forward | TGGCAGTAATGCTGGTTGTGA | 98 |
|  | Reverse | GCTGGGCTGGAGAAAGGAC |  |
| *Nis* | Forward | CTGTGGCATTGTCATGTTCGT | 122 |
|  | Reverse | CTCCGGGCAGATCCTCAAA |  |
| β-Actin | Forward | CTAAGGCCAACCGTGAAAAGA | 99 |
|  | Reverse | CCAGAGGCATACAGGGACAAC |  |

Tg, thyroglobulin; Tpo, thyroperoxidase; Nis, sodium iodide symporter
